# *TRIM5* Suppresses Cross-Species Transmission of a Primate Immunodeficiency Virus and Selects for Emergence of Resistant Variants in the New Species

**DOI:** 10.1371/journal.pbio.1000462

**Published:** 2010-08-24

**Authors:** Andrea Kirmaier, Fan Wu, Ruchi M. Newman, Laura R. Hall, Jennifer S. Morgan, Shelby O'Connor, Preston A. Marx, Mareike Meythaler, Simoy Goldstein, Alicia Buckler-White, Amitinder Kaur, Vanessa M. Hirsch, Welkin E. Johnson

**Affiliations:** 1 New England Primate Research Center, Department of Microbiology and Molecular Genetics, Harvard Medical School, Southborough, Massachusetts, United States of America; 2 Institut für Klinische und Molekulare Virologie, Friedrich-Alexander-Universität Erlangen-Nürnberg, Germany; 3 Laboratory of Molecular Microbiology, National Institute of Allergy and Infectious Disease, National Institutes of Health, Bethesda, Maryland, United States of America; 4 Genome Sequencing and Analysis Program, Broad Institute of MIT and Harvard, Cambridge, Massachusetts, United States of America; 5 Department of Pathology and Laboratory Medicine, University of Wisconsin-Madison, Madison, Wisconsin, United States of America; 6 Tulane Regional Primate Research Center, Covington, Louisiana, United States of America; 7 New England Primate Research Center, Division of Immunology, Harvard Medical School, Southborough, Massachusetts, United States of America; Fred Hutchinson Cancer Research Center, United States of America

## Abstract

Simian immunodeficiency viruses of sooty mangabeys (SIVsm) are the source of multiple, successful cross-species transmissions, having given rise to HIV-2 in humans, SIVmac in rhesus macaques, and SIVstm in stump-tailed macaques. Cellular assays and phylogenetic comparisons indirectly support a role for TRIM5α, the product of the *TRIM5* gene, in suppressing interspecies transmission and emergence of retroviruses in nature. Here, we investigate the in vivo role of *TRIM5* directly, focusing on transmission of primate immunodeficiency viruses between outbred primate hosts. Specifically, we retrospectively analyzed experimental cross-species transmission of SIVsm in two cohorts of rhesus macaques and found a significant effect of *TRIM5* genotype on viral replication levels. The effect was especially pronounced in a cohort of animals infected with SIVsmE543-3, where *TRIM5* genotype correlated with approximately 100-fold to 1,000-fold differences in viral replication levels. Surprisingly, transmission occurred even in individuals bearing restrictive *TRIM5* genotypes, resulting in attenuation of replication rather than an outright block to infection. In cell-culture assays, the same *TRIM5* alleles associated with viral suppression in vivo blocked infectivity of two SIVsm strains, but not the macaque-adapted strain SIVmac239. Adaptations appeared in the viral capsid in animals with restrictive *TRIM5* genotypes, and similar adaptations coincide with emergence of SIVmac in captive macaques in the 1970s. Thus, host *TRIM5* can suppress viral replication in vivo, exerting selective pressure during the initial stages of cross-species transmission.

## Abstract 2

Cross-species transmission of simian immunodeficiency virus from sooty mangabeys (SIVsm) into rhesus macaques, and subsequent emergence of pathogenic SIVmac, required adaptation to overcome restriction encoded by the macaque *TRIM5* gene.

## Author Summary

The human immunodeficiency viruses HIV-1 and HIV-2 originated from cross-species transmission of simian immunodeficiency viruses (SIVs) from chimpanzees (SIVcpz) and sooty mangabeys (SIVsm), respectively. A related virus, SIVmac, causes AIDS-like pathogenesis in rhesus macaques; like HIV-2, SIVmac is the product of a cross-species jump of SIVsm from sooty mangabeys. The primate *TRIM5* gene encodes a factor with potent antiviral activity when tested in the laboratory, and TRIM5 proteins are thought to play a role in restricting the movement of viruses between species in nature. In this study, we show that genetic variation in the *TRIM5* gene of rhesus macaques heavily influences the outcome of cross-species transmission of SIVsm and that emergence of SIVmac in rhesus macaques in the 1970s required adaptations to circumvent the genetic barrier imposed by the rhesus macaque *TRIM5* gene. Our results confirm the hypothesis that *TRIM5* can influence the process of cross-species transmission and emergence of viruses related to HIV-1 and HIV-2 and serve as a striking illustration of how host genes can influence virus evolution.

## Introduction

The Simian immunodeficiency viruses (SIVs) are widespread among African primates [1]. However, host and viral phylogenies are not completely congruent; such a pattern argues against co-divergence of virus and host lineages since the time of a common, infected primate ancestor and argues instead that the modern distribution of SIVs among extant primates resulted, at least in part, from cross-species transmission events followed by emergence of new virus/host combinations [2]. The most notable examples include cross-species transmission of SIV from apes to humans, which gave rise to HIV-1 and initiated the worldwide AIDS epidemic, and cross-species transmission of SIV from sooty mangabeys (SIVsm) to humans, which gave rise to the more limited HIV-2 epidemic [1],[3],[4]. In a striking parallel to the emergence of HIV-1 and HIV-2, SIVsm also jumped into captive Asian macaques in the United States, resulting in emergence of SIVmac and outbreaks of AIDS-like disease at several U.S. National Primate Research Centers in the 1970s [3],[5],[6]. The exact time and means by which SIVsm was transmitted to macaques are unknown, but since isolation of the first SIV strains from captive macaques in the 1980s, experimental infection of rhesus macaques with SIV has become the primary animal model for preclinical research on AIDS vaccines and pathogenesis. Variation in susceptibility to infection and disease progression in nonhuman primate models often confounds such studies, and identifying the sources of variation will lead to more efficient use of AIDS models. At the same time, genetic variation in nonhuman primate hosts of SIV provides unique and powerful opportunities to study the impact of host genetics on cross-species transmission, adaptation, and emergence of viruses. In the present study, we establish that allelic variation in the rhesus macaque *TRIM5* gene results in differences in susceptibility to infection and viral replication in the early stages of cross-species transmission of SIVsm and that emergence of pathogenic SIVmac in rhesus macaques required adaptations in the viral capsid protein (CA) to overcome suppression by two distinct types of *TRIM5* allele.

Viral inhibition by TRIM5α, a product of the *TRIM5* gene, is initiated by an interaction between the protein's B30.2/SPRY domain and virion capsid-cores released into the target-cell cytoplasm after viral attachment and entry [7]–[13]. The target of TRIM5α involves the N-terminal domain (NTD) of the viral CA protein [14]. We previously discovered that rhesus macaque *TRIM5* is highly polymorphic, including eight nonsynonymous polymorphisms tightly clustered in the B30.2/SPRY domain [15]. One of these, a six-nucleotide insertion/deletion, results in a TFP/Q length polymorphism. When tested against multiple lentiviruses, TFP^339-341^ and Q^339^ alleles (*TRIM5^TFP^* and *TRIM5^Q^*) give different patterns of restriction [16]. An unusual haplotype encoding a TRIM5-cyclophilin-A chimera (TRIM5CypA) is also found among rhesus macaques [17],[18]. The chimeric TRIM5CypA lacks a B30.2/SPRY domain and in its place encodes a CypA domain derived from a retrotransposed CypA reading frame inserted in the 3′UTR [17]–[21]. TRIM5CypA restriction of lentiviral infection involves specific binding to a peptide loop between helices 4 and 5 of the viral CA (also the binding site for cellular CypA) [22],[23].

## Results

**Figure 1:**
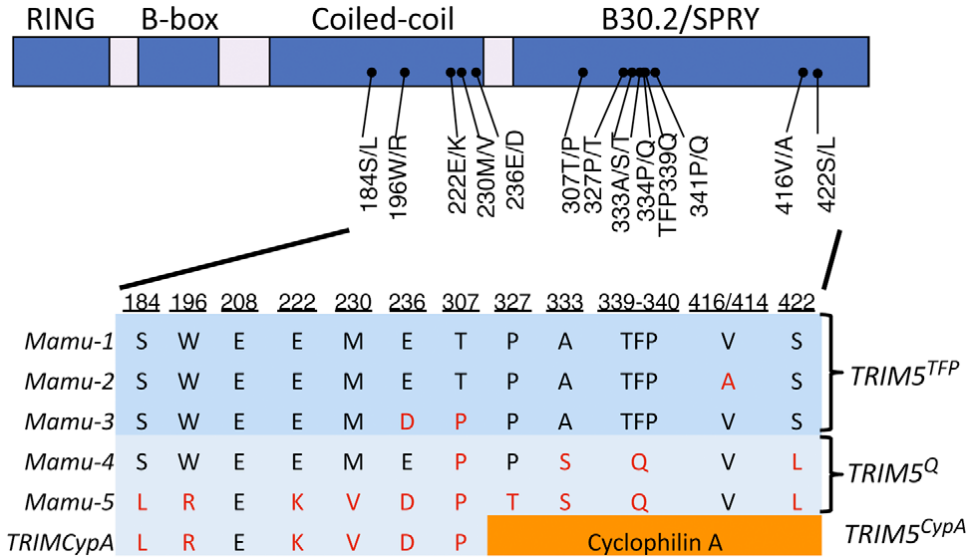
The rhesus macaque *TRIM5* coding sequence is highly polymorphic. Rhesus macaque TRIM5 proteins are distinguished by polymorphic variation in the B30.2/SPRY domain (encoded by *TRIM5^TFP^* and *TRIM5^Q^* alleles), or by complete replacement of the B30.2/SPRY domain with a CypA domain (encoded by the *TRIM5^CypA^* allele). Shown are polymorphic sites that differentiate six common rhesus *TRIM5* alleles, including three *TRIM5^TFP^* alleles (Mamu-1, Mamu-2, and Mamu-3), two *TRIM5^Q^* alleles (Mamu-4, Mamu-5), and the *TRIM5CypA* allele. For more details, see references [15],[17]. Accession numbers for the depicted alleles are also available in GenBank as follows: Mamu-1 through Mamu-5 (EF113914–EF113918); *TRIM5CypA* (EU359036).

Common variants of rhesus *TRIM5* can be grouped into three allelic classes: *TRIM5CypA*, *TRIM5^TFP^*, and *TRIM5^Q^* (Figure 1) [16],[24]. We established the existence of all six possible genotypes in a large colony of captive rhesus macaques, using archived genomic DNA samples from the Genetics Core of the New England Primate Research Center. In this colony, we observed frequencies of 46% (*TRIM5^TFP/TFP^*), 36% (*TRIM5^TFP/Q^*), 5% (*TRIM5^TFP/CypA^*), 10% (*TRIM5^Q/Q^*), 1% (*TRIM5^Q/CypA^*), and 2% (*TRIM5^CypA/CypA^*). These values indicate allele frequencies in one particular colony of rhesus macaques; because of differences in animal husbandry practices and potential founder effects, these values do not necessarily reflect the distribution of genotype frequencies within other captive colonies or in wild rhesus monkey populations. Nonetheless, the presence of allelic variation in the rhesus *TRIM5* gene can be exploited to study the impact of *TRIM5* expression in vivo.

To ask whether TRIM5-mediated restriction plays a role in cross-species transmission and emergence of primate lentiviruses, we tested six representative alleles of rhesus macaque *TRIM5* for restriction activity against four closely related viruses of old-world monkeys, SIVmac239, SIVsmE543, SIVsmE041, and SIVstm/37.16 (Table 1). SIVmac239 is a molecular clone of a highly adapted, emergent virus of rhesus macaques [25], generated in the 1980s by experimental passage of SIV-positive plasma through a series of five monkeys [26]. In all likelihood, SIVmac239 is descended from a cross-species transmission event that took place in the 1960s in captive colonies of rhesus macaques [5], probably as the unintended consequence of experiments involving transfer of biological material from SIVsm-positive sooty mangabeys to rhesus macaques [27]. Regardless of origin, as a result of this long association with macaques, experimental infection with SIVmac239 reproducibly results in high levels of persistent viral replication [28]. In contrast, SIVsmE543-3 is a molecular clone derived by intentional inoculation of a rhesus macaque with plasma from an SIVsm-infected sooty mangabey, followed by passage through one additional rhesus macaque [29]; thus, opportunity for SIVsmE543-3 to adapt to macaques was limited to only two animals. As a result, SIVsmE543-3 replication in macaques is highly variable, with acute viral loads ranging from 10^3^ to 10^8^ viral RNA copies/ml plasma, and set-point values from <100 to 10^8^. Variation in SIVsmE543-3 infected animals is consistent with an influence of genetic variation in a host gene or genes [30]. SIVsmE041 is a biological isolate cultured directly from an SIV-positive sooty mangabey [31] and has therefore not experienced any prior adaption to rhesus macaques. SIVstm/37.16 is an SIV isolate from a different species, the stump-tailed macaque (*M. arctoides*), and represents an independent cross-species transmission event involving transmission of SIVsm directly to *M. arctoides* animals [3],[27],[32],[33]. The relevant properties of these four viruses are summarized in Table 1.

**Table 1:** Overview of SIV strains used in this study.

Virus	Passage History	Phenotype in Rhesus Macaques	Reference
SIVsmE041	primary sooty mangabey isolate	low pathogenicity; variable viremia	[31],[34]
SIVsmE543-3	cloned after experimental passage of SIVsm through two rhesus macaques	variable pathogenicity; variable viremia	[29]
SIVmac239	descendant of SIVsm; history in rhesus macaques uncertain (>10 y)	high pathogenicity; high viremia	[25],[27],[28]
SIVstm/37.16	descendant of SIVsm; history in stump-tailed macaques uncertain (>10 y)	unknown	[32],[33]

**Figure 2:**
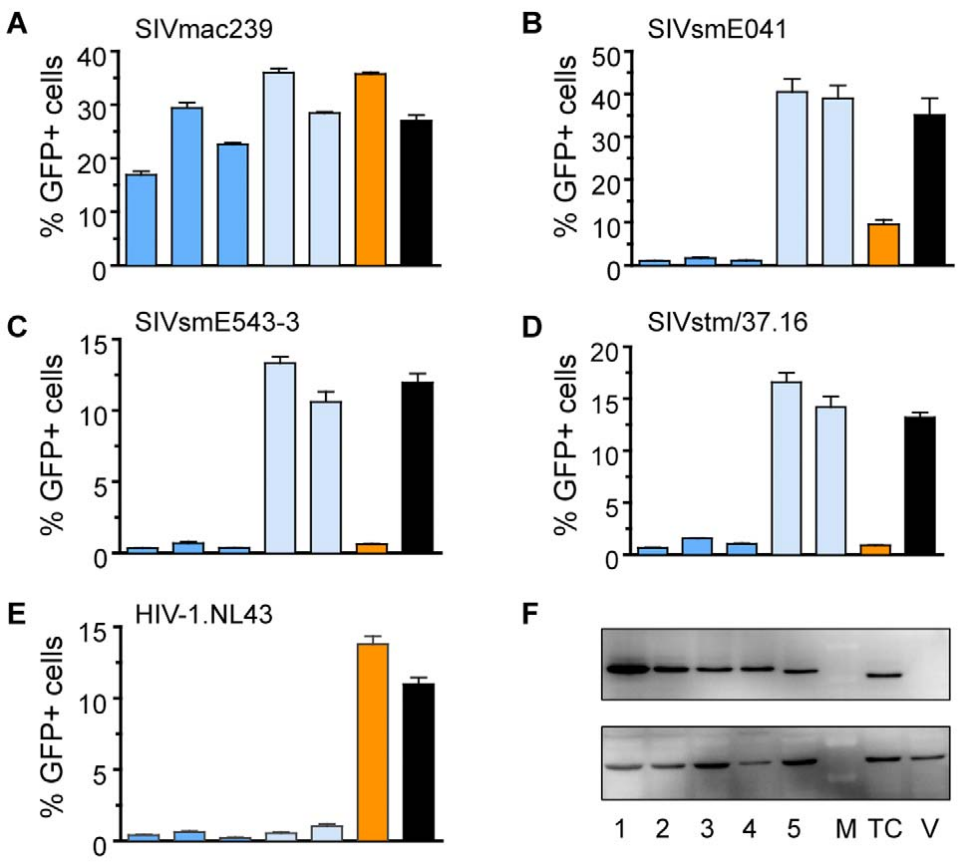
Differential restriction of SIVmac and SIVsm strains by multiple alleles of rhesus macaque *TRIM5*. Single-cycle infectivity was measured on a panel of cell lines stably expressing six common alleles of rhesus *TRIM5* [15],[17]. Alleles tested included three *TRIM5^TFP^* (*Mamu*-1, *Mamu*-2, *Mamu*-3, dark blue bars), two *TRIM5^Q^* (*Mamu*-4 and *Mamu*-5, light blue bars), and *TRIM5CypA* (orange bars). Control cells stably expressing the empty vector served as a negative control (black bars). Of the four closely related SIVs, only the rhesus macaque isolate SIVmac239 is resistant to multiple alleles. Infectivity (% GFP-positive cells) was measured by flow-cytometry (error bars indicate ± SEM). Virus stocks were first titered by serial dilution on parental cells and normalized (Figure S1). Stable cell lines were generated from CRFK cells as described in the Materials and Methods section. HIV-1NL4-3 was used as a positive control to confirm expression and function of the *TRIM5^Q/Q^* alleles. (A) SIVmac239; (B) SIVsmE041; (C) SIVsmE543-3; (D) SIVstm37/13; (E) HIV-1NL4-3; (F) Immunoblot confirming expression of HA-tagged TRIM5 proteins in cell lysates. *upper panel*, TRIM5 proteins; *lower panel*, β-actin loading control. Lanes: (1) Mamu-1; (2) Mamu-2; (3) Mamu-3; (4) Mamu-4; (5) Mamu-5; M, protein standard; TC, TRIM5CypA; V, control cells expressing vector-only. GenBank accession number for the SIVsmE041 clone: HM059825.

Infectivity of all four viruses was measured on cell lines stably expressing six common alleles of rhesus macaque *TRIM5*, including three *TRIM5^TFP^* alleles, two *TRIM5^Q^* alleles, and the *TRIM5^CypA^* allele (Figure 2). SIVmac239 was resistant to all six. SIVsmE041, SIVsmE543, and SIVstm were also resistant to *TRIM5^Q^* but unlike SIVmac239 were sensitive to both *TRIM5^CypA^* alleles and *TRIM5^TFP^* alleles (Figure 2B,C,D). Of the four SIV strains tested, only SIVmac239 is the product of decades of replication and spread in rhesus macaques. Thus, the comparison suggests that sensitivity to *TRIM5^TFP^* and *TRIM5^CypA^* alleles represents the ancestral phenotype and that emergence of SIVsm (as SIVmac) in rhesus macaques required acquisition of adaptive changes to overcome those particular types of alleles. In contrast, the SIVsm variants that first invaded rhesus macaques were probably inherently resistant to *TRIM5^Q^* alleles.

**Figure 3:**
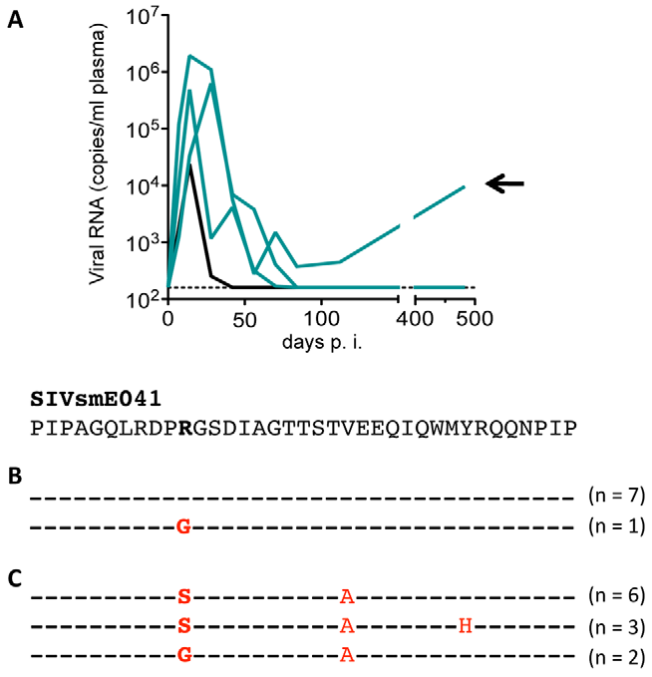
Attenuated replication of the sooty mangabey virus SIVsmE041 upon experimental cross-species transmission to rhesus macaques. The cohort was described previously [34]; briefly, four Indian-origin rhesus macaques were inoculated intravenously with 25 ng p27 equivalent of an SIVsmE041 virus stock derived by co-culture of cells from SIV-infected sooty mangabey #E041 on PBMC from a second, SIV-negative sooty mangabey. Viral RNA levels in plasma (*y*-axis) were determined by quantitative RT-PCR at different time-points post-infection (*x*-axis). All four rhesus macaques displayed post-acute reduction of viral replication by several orders of magnitude. Genotypes were *TRIM5^TFP/TFP^* (3 animals, green lines) and *TRIM5^TFP/CypA^* (one animal, black line). The three homozygotes had acute viral RNA levels peaking between 10^5^ and 10^6^ (RNA copy equivalents/ml of plasma), whereas the heterozygote had the lowest acute viral RNA levels (2.3×10^4^ RNA copy eqs/ml). Viral replication rebounded in one of the *TRIM5^TFP/TFP^* animals (black arrow), suggestive of adaptation and escape. (A) Viral RNA levels in plasma. (B) Partial sequencing of the region encoding the N-terminal domain of the viral capsid from the indicated animal (black arrow), using samples collected during acute infection. (C) Partial sequencing of the region encoding the N-terminal domain of the viral capsid from the indicated animal (black arrow) using samples collected during week 89 post-infection. Comparison of (B) and (C) revealed potential adaptive changes at amino-acid positions 97 and 108 (SIVmac239 numbering).

To analyze the impact of *TRIM5* variation on cross-species transmission directly, we acquired samples from two independent SIVsm/macaque cohorts. The smaller cohort consisted of four sooty mangabeys and four rhesus macaques that had been experimentally inoculated with SIVsmE041 [34]. Infection of sooty mangabeys (natural hosts of SIVsm) resulted in ready take of virus and persistent infection (unpublished data), whereas infection of macaques resulted in a transient infection followed by a decrease in viral replication to a point near or below the detection limit (Figure 3). Using archived DNA, we determined that the four macaques included three *TRIM5^TFP/TFP^* homozygotes and one *TRIM5^TFP/CypA^* heterozygote. Thus, the alleles present in these four animals were all of the same types that restricted SIVsmE041 in tissue culture (Figure 2B). In one animal, replication resurged during the first year, reaching ∼10^4^ RNA copies/ml plasma (Figure 3). Capsid sequences recovered from this animal revealed the appearance of two fixed changes, R97S and V108A (SIVmac239 numbering) at the late time point (Figure 3C). In contrast, amplification and sequencing from acute infection, from the SIVsmE041 inoculum, and from an infected sooty mangabey, revealed only the ancestral states at both positions (R97 and V108) (Figure 3B).

**Figure 4:**
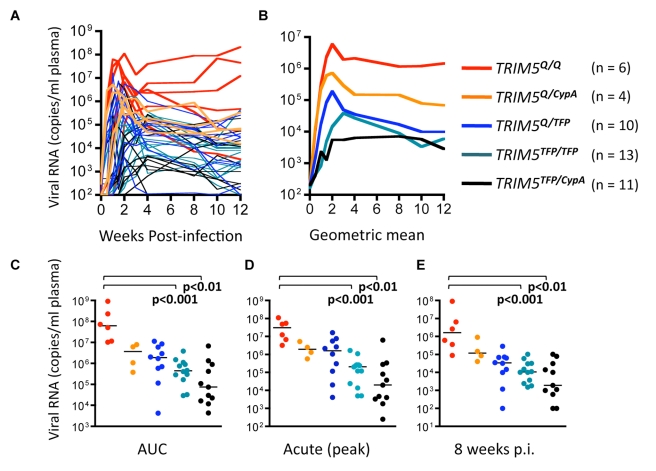
*TRIM5* genotype and replication of SIVsmE543-3 in rhesus macaques. Archived samples were obtained from 43 Indian origin rhesus macaques that had been infected intravenously (*n* = 35) or intrarectally (*n* = 9) with SIVsmE543-3 (TCID 50% from 1 to 1,000). None of the animals were treated or vaccinated prior to infection. Genomic DNA extracted from stored PBMC was used to determine *TRIM5* genotype. Data are color-coded by genotype, as follows: red, *TRIM5^Q/Q^*; orange, *TRIM5^Q/CypA^*; blue, *TRIM5^TFP/Q^*; green, *TRIM5^TFP/TFP^*; and black, *TRIM5^TFP/CypA^*. (A) Viral replication (RNA copy equivalents per milliliter of plasma) through 12 wk post-infection; (B) Same data presented as geometric mean values for each genotype; (C) area under the curve; (D) acute infection (defined as peak viremia for each animal during the first 4 wk post-infection); (E) 8 wk post-infection. Viral replication levels were compared by non-parametric one-way ANOVA (Kruskal-Wallis test) with Dunn's post-test. Pairs of groups that differed significantly are indicated (*p*<0.01 or *p*<0.001).

The second and larger cohort consisted of historical samples from 44 SIVsmE543-3-infected rhesus macaques. Genotype frequencies in this cohort were 30% *TRIM5^TFP/TFP^*, 23% *TRIM5^TFP/Q^*, 26% *TRIM5^TFP/CypA^*, 12% *TRIM5^Q/Q^*, 9% *TRIM5^Q/CypA^*, and 0% *TRIM5^CypA/CypA^*. Animals with two restrictive alleles (*TRIM5^TFP/TFP^* and *TRIM5^TFP/CypA^*) had dramatically diminished viral replication compared to *TRIM5^Q/Q^* homozygotes, with mean (geometric) differences of 830-fold and 1,728-fold, respectively, by 8 wk post-infection (Figure 4). Animals with one restrictive allele (*TRIM5^TFP/Q^* and *TRIM5^CypA/Q^* heterozygotes) displayed intermediate levels of viral replication. Taken in conjunction with the clear differences in restriction of SIVsmE543-3 by *TRIM5^TFP^*, *TRIM5^CypA^*, and *TRIM5^Q^* in vitro (Figure 2C), these results are consistent with allelic variation in *TRIM5* having a significant impact on SIVsmE543 replication kinetics in rhesus macaques. We also obtained archived DNA from animal #E543, the source of the original SIVsmE543-3 clone [29], and determined that this animal had been a *TRIM5^Q/Q^* homozygote. The fact that E543 bore a non-restrictive genotype may well have facilitated isolation of the original SIVsmE543-3 clone.

Several reports describe a correlation between specific alleles of class I *MHC* and enhanced control of SIVmac239/SIVmac251 infection in rhesus macaques [35]–[37]. Specifically, *MHC* class-I Mamu-B*08 and Mamu-B*17 alleles have been associated with lower levels of chronic phase viral replication in SIVmac239-infected animals [35],[37]. Several observations argue against the dramatic differences in viral replication of SIVsmE543-3 in rhesus macaques being due to Mhc class I rather than TRIM5. First, the *TRIM5* and class I *MHC* loci are on different chromosomes, reducing the probability of a chance association between suppressive alleles of *TRIM5* and a specific allele or alleles of class I *MHC* with activity against SIVsmE543-3. More importantly, the effects of class I Mhc on SIVmac239 replication do not manifest during the acute stage of infection [38], whereas the correlation with *TRIM5* genotype is already apparent during acute infection (Figure 4D). Finally, Goldstein et al. demonstrated that variation in susceptibility of cells taken from naïve animals (prior to infection) and tested ex vivo correlated with viral replication levels in vivo, when the same animals were subsequently infected with SIVsmE543; such results argue in favor of an inherent, genetic cause of variation in susceptibility and against an effect of virus-specific adaptive immune responses induced by infection [30]. Finally, we typed all 43 animals in the SIVsm543-3 cohort for the presence of the Mamu-B*08 and Mamu-B*17 alleles and found no association between these alleles and the observed differences in Figure 4. Most importantly, none of the infected animals with *TRIM5^Q/Q^* or *TRIM5^TFP/CypA^* genotypes were Mamu-B*08 or Mamu-B*17 positive, so the statistically significant differences between those two groups cannot be attributed to Mamu-B*08 or Mamu-B*17 associated control of SIVsmE543. Thus, the observed correlation between *TRIM5* genotype and SIVsmE543-3 replication levels is not due to a spurious association between suppressive alleles of *TRIM5* and class I *MHC* alleles previously associated with control of SIVmac239. It is important to note, however, that this result does not rule out a general influence of class I Mhc on viral replication levels in rhesus macaques, only that *MHC* genotype does not explain the correlation depicted in Figure 4. Among other things, allelic variation in *MHC* class I may well contribute to the significant variation observed within groups (Figure 4C,D,E).

**Figure 5:**
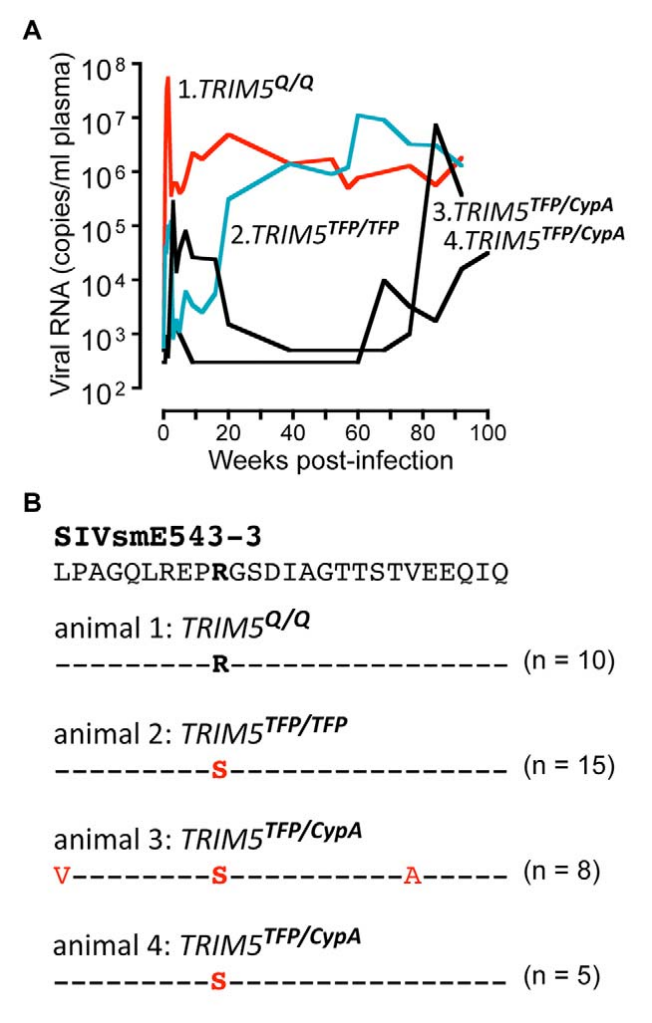
Emergence of Trim5-resistant SIV in vivo. (A) Viral replication suggestive of suppression followed by adaptation in three animals, as indicated by a green line (*TRIM5^TFP/TFP^* homozygote) and two black lines (*TRIM5^TFP/CypA^* heterozygotes). (B) Sequences encoding a portion of the N-terminal region of CA were amplified, cloned, and sequenced from all three animals, as well as from a *TRIM5^Q/Q^* homozygote with typical, high levels of persistent viral replication. Also shown is the sequence of the SIVsmE543-3 clone. An R97S change was present in every clone from the three animals with restrictive genotypes, but not in the non-restrictive *TRIM5^Q/Q^* control animal. At the nucleotide level, both Arg(AGA)-to-Ser(AGC) and Arg(AGA)-to-Ser(AGT) were seen. The aligned amino-acid sequences correspond to residues 89–113 in the N-terminal domain of the SIVmac239 capsid; however, SIVsmE543-3 has one additional amino-acid in this region compared to SIVmac239, so that R97S actually appears at position 98 in the depicted alignment.

Three macaques in the SIVsmE543-3 cohort also had patterns of resurgent viral replication consistent with escape from suppression (Figure 5A). All three animals had two restrictive alleles and included one *TRIM5^TFP/TFP^* homozygote and two *TRIM5^TFP/CypA^* heterozygotes. We amplified *gag* sequences encoding the NTD of CA from all three animals, and from a *TRIM5^Q/Q^* animal, and compared these to the original SIVsmE543-3 clone (Figure 5B). Strikingly, an R97S change was present in every clone from all three animals, typically due to an AGA->AGC substitution, although 3/15 clones in one animal were AGA->AGT. No changes were found in the corresponding region of virus from the non-restrictive *TRIM5^Q/Q^* animal. Thus, an identical R97S change appeared independently in four animals with suppressive *TRIM5* genotypes, including three SIVsmE543-infected animals (Figure 5B) and one SIVsmE041-infected animal (Figure 3).

**Figure 6:**
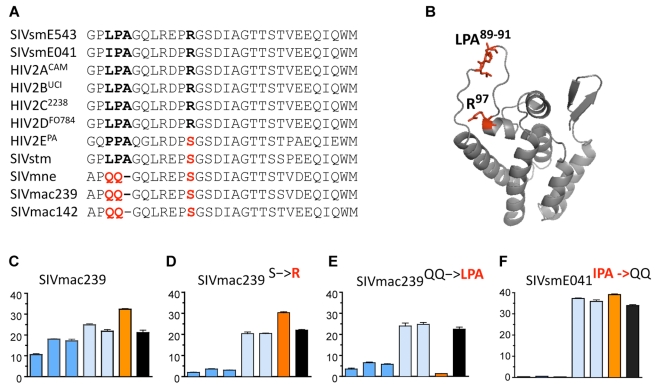
Adaptations in the CA of SIVmac strains confer resistance to a subset of rhesus *TRIM5* alleles. (A) Partial alignment of the NTD of CA from multiple primate lentiviruses highlights the unusual QQ^89,90^ sequence at the tip of the CypA-binding loop, which is unique to the SIVmac lineage. Also shown is an inferred change at position 97 at the base of the loop (in helix 5), identical to the R97S change that arose in the SIVsm experimental cohorts shown in Figures 2 and 3. (B) Location of LPA^89–91^ and R^97^ on the HIV-2 CA crystal structure, highlighted in red (structure is from reference [23]). (C) Infectivity of parental SIVmac239; bars are color-coded as described in the legend of Figure 2. (D) Infectivity of SIVmac239^S97R^, in which S97 has been reverted to the ancestral R97, reveals a gain of sensitivity to *TRIM5^TFP^* alleles (dark blue bars). (E) Infectivity of SIVmac239^QQ/LPA^ (in which QQ^89,90^ has been reverted to the ancestral LPA^89–91^) reveals a gain of sensitivity to *TRIM5^TFP^* alleles (dark blue bars) and *TRIM5^CypA^* (orange bars). (F) Changing the ancestral IPA to QQ in SIVsmE041 (SIVsmE041^IPA/QQ^) results in a gain of resistance to TRIM5CypA.

Phylogenetic analyses are consistent with a minimum of two historical transmissions of SIVsm into macaques, one into stump-tailed macaques (SIVstm), and the other into rhesus macaques (SIVmac); both transmissions are thought to have occurred in captive macaques sometime prior to the 1970s [3]. If TRIM5-mediated restriction influences cross-species transmission of primate lentiviruses, we predict the existence of adaptive changes in SIV isolates corresponding to such events. Indeed, alignment of the NTD of several lentiviruses in the SIVsm/SIVmac/HIV-2 lineage revealed multiple potential adaptations in SIVmac (Figure 6). Most striking is an inferred R97S change, identical to the change that appeared in the SIVsmE041 and SIVsmE543 experimental cohorts described above (Figures 3 and 5B). Based on phylogeny of the SIVsm/SIVmac/HIV-2 lineage [3], the R97S change was probably selected twice, once coinciding with emergence of SIVmac in rhesus macaques and once coinciding with emergence of SIVstm in stump-tailed macaques. Consistent with this interpretation, the underlying nucleotide substitutions are different in the two viruses (AGA->AGC in SIVstm, and AGA->TCA in SIVmac). However, S97 is also found in a small percentage of HIV-2 and SIVsm isolates; thus, it is possible that one or both historical transmissions were initiated by an SIVsm with serine at position 97, rather than de novo mutation as seen in the experimental cohorts. In either case, these combined observations strongly suggest that S97 is selectively advantageous in macaques.

The second putative adaptation is a highly unusual LPA/QQ substitution at the tip of the CA 4–5 loop (QQ^89,90^ in SIVmac239). How this change was generated is unclear but must have involved multiple point mutations and a net loss of three nucleotides. While QQ^89,90^ is very common among SIVmac isolates, P^90^ (the proline in LPA^89–91^) is extremely well conserved in SIVsm and HIV-2 isolates (Figure 6). The highly unusual nature of the LPA->QQ substitution, together with its location in a stretch of residues known to affect TRIM5-mediated restriction [14],[39], mark it as a potential adaptation that arose to circumvent restriction by rhesus macaque TRIM5 proteins.

To ask whether the R97S and LPA/QQ changes in SIVmac strains arose as adaptations to overcome rhesus TRIM5, we reverted these sites to the ancestral sequence in the context of the macaque-adapted strain SIVmac239. We then tested SIVmac239^S97R^ and SIVmac239^QQ->LPA^ for gain-of-sensitivity to different rhesus *TRIM5* alleles. Indeed, the S97R reversion resulted in increased sensitivity to all three *TRIM5^TFP^* alleles but had no effect on resistance to the *TRIM5^Q^* or *TRIM5CypA* alleles. In contrast, the QQ^89,90^-to-LPA^89-91^ reversion resulted in sensitivity to both *TRIM5^TFP^* and *TRIM5CypA* alleles. This closely resembles the pattern displayed by SIVsm isolates (see Figure 2B and C), confirming that these represent bona fide adaptations to overcome restriction by rhesus *TRIM5* alleles. Because QQ^89,90^ is in the 4–5 loop of capsid, the adaptation probably functions by altering the TRIM5CypA binding site. The structural basis for the interaction between the TRIM5 B30.2/SPRY domain and viral CA is not yet well defined, and it is not clear why QQ^89,90^ also affects resistance to *TRIM5^TFP^* alleles. However, this result is consistent with studies showing that site-directed mutations in the HIV-1 CypA-binding domain influence binding and restriction by TRIM5α proteins [39]. A better understanding of the influence of CA positions 89–91 on restriction awaits more detailed, structural understanding of the interaction between CA and TRIM5α.

Neither reversion affected resistance of SIVmac239 to *TRIM5^Q^*, consistent with our observation that SIVsmE041 and SIVsmE543, which retain ancestral states at these sites, were also resistant to *TRIM5^Q^* (Figure 2). We conclude that *TRIM5^Q^* alleles did not significantly hamper SIVsm colonization of rhesus macaques or its emergence as SIVmac in the 1970s. In fact, *TRIM5^Q/Q^* animals may have facilitated initial transmission of SIVsm among macaques, permitting higher levels of replication and increasing the probability of adaptation and spread.

Unlike the R97S change, we did not see the LPA^89–91^/QQ^89,90^ change appear in either experimental cohort (Figures 3 and 5B), possibly because the multiple mutations involved make it a low probability occurrence. Once virus with the QQ^89,90^ motif in CA appeared, however, it probably contributed to emergence of SIVmac by facilitating spread among animals bearing restrictive *TRIM5^CypA^* alleles. Consistent with this hypothesis, experimental introduction of the QQ^89,90^ sequence at the homologous positions in the sooty mangabey strain SIVsmE041 by site-directed mutagenesis rendered the virus resistant to TRIM5CypA (Figure 6). The mutation did not affect sensitivity to *TRIM5^TFP^* alleles or resistance to *TRIM5^Q^* alleles. Thus, reversion to the ancestral state in SIVmac239 (changing QQ^89,90^ to LPA^89–91^) resulted in sensitivity to restriction by TRIM5CypA, and the reciprocal substitution to recreate the evolutionarily derived state in SIVsmE041 (IPA to QQ) resulted in resistance to TRIM5CypA. These results are consistent with the hypothesis that the QQ^89,90^ sequence is an adaptation to overcome rhesus TRIM5CypA that arose during emergence of the SIVmac lineage. In the case of SIVmac239^QQ/LPA^, the reversion also affected resistance to *TRIM5^TFP^* alleles, suggesting that the effects of the adaptation may be influenced by additional differences between the SIVsm and SIVmac capsids. Finally, note that two mutants, SIVsmE041^IPA->QQ^ and SIVmac239^S97R^, have similar patterns of restriction (compare Figure 6B and 6D), consistent with the fact that these two viruses are identical at the two sites in question (i.e., both viruses are QQ^89,90^ and R^97^).

## Discussion

Host-encoded, dominant-acting blocks to retroviral infection, or restriction factors (RF), were first defined genetically for murine and avian retroviruses more than 40 years ago [40]. Over the last decade, experiments seeking the causes of defined cellular blocks to HIV-1 infection uncovered three new RF genes, *APOBEC3G* (now considered to be the prototype of a cluster of ∼8 *APOBEC3* genes), *TRIM5*, and *Tetherin* [11],[41],[42]. Consistent with the notion that these genes played a role in protecting host organisms from retroviral infections during the course of mammalian evolution, *Tetherin*, *APOBEC3G*, and *TRIM5* display signs of long-term positive selection, including high levels of amino-acid diversification between closely related species [9],[43]–[46]. It is generally assumed that RF genes such as *TRIM5*, *APOBEC3G*, and *Tetherin* influence the distribution and spread of viruses between hosts, with viral epidemics resulting in selective sweeps of these loci, and successful viruses in turn adapting to the spectrum of restriction encoded by each host species [47]. Our results provide direct evidence that expression of one of these factors (TRIM5) can indeed suppress viral replication during the early stages of cross-species transmission and that under such conditions, viral emergence and pathogenesis in the new host species requires adaptation to overcome restriction. Given overall similarities in evolutionary patterns and biological function, it seems very likely that the APOBEC3 enzymes and Tetherin/BST2 will also be found to influence patterns of cross-species transmission and viral emergence among extant species.

In this study, we specifically demonstrated that the RF gene *TRIM5* can suppress replication of a primate immunodeficiency virus in vivo at the time of cross-species transmission, and that TRIM5-mediated suppression of viral replication selects for acquisition of adaptive changes in the viral capsid protein. The data included retrospective genotyping of two independent, cross-species transmission cohorts, identification of at least two adaptations selected for resistance to TRIM5 during emergence of the SIVmac lineage, and functional assessment of allele-specific restriction in cell-culture. While the adaptations in SIVmac239 capsid identified here (R^97^S and LPA^89–91^/QQ^89,90^) have a direct effect on restriction sensitivity, the modification of the otherwise highly conserved residues R97 and P90 may well have required additional adaptive adjustments elsewhere in the CA or the Gag polyprotein. Identifying such changes, if they exist, will require systematic testing of combinations of residues that vary between the SIVmac and SIVsm lineages for impact on relative infectivity in the presence and absence of *TRIM5* expression.

**Figure 7:**
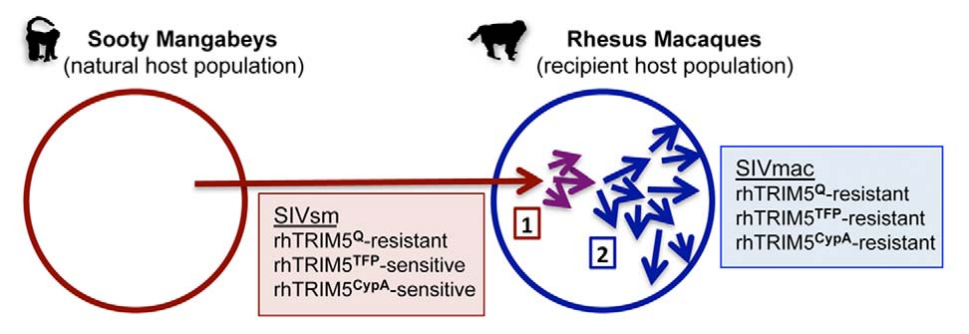
Scenario depicting the inferred role of rhesus macaque *TRIM5* in emergence of SIV in captive Asian macaque colonies in the late 20th century. Colored circles represent hypothetical populations of sooty mangabey monkeys (red circle) and rhesus macaques (blue circle). In this scenario, at the time of transmission, SIVsm was initially resistant to rhesus *TRIM5^Q^* alleles but sensitive to alleles of the *TRIM5^TFP^* and *TRIM5^CypA^* types. Initially (1), Infection of animals bearing *TRIM5^Q^* alleles (particularly *TRIM5^Q/Q^ animals)* permitted high-titer, persistent infection, and the greatest potential for further transmission in the new host species. Replication in these animals also provided opportunity for adaptation to the new host. Subsequently (2), the appearance of adaptive changes in the N-terminal domain of the viral capsid protein (including S^97^ and QQ^89,90^) allowed viral replication in a larger percentage of the population and ultimately facilitated emergence of pathogenic SIVmac.

The transmission event(s) leading to spread and emergence of SIVsm in the U.S. macaque colonies have not been identified, although there is indirect evidence suggesting that transmission may have resulted from experimental procedures involving the transfusion of material from one species into another [27]. Regardless of the mechanism(s) of initial transmission, our data strongly suggest that adaptation to overcome restriction by a specific subset of rhesus macaque *TRIM5* alleles played a key role in spread of SIVsm and its emergence as SIVmac in captive colonies of rhesus macaques. Based on cell-culture assays, analysis of experimental cohorts, and functional identification of adaptive changes in the SIVmac239 capsid, we propose a simple but likely scenario for the influence of variation in the rhesus macaque *TRIM5* gene on emergence of SIVmac in the U.S. National Primate Research Center macaque colonies (Figure 7). Briefly, at the time of initial cross-species exposure, we believe that the SIVsm source was likely to be sensitive to restriction by a subset of rhesus macaque *TRIM5* alleles, including those with a TFP sequence at residues 339–341 in the B30.2/SPRY domain (*TRIM5^TFP^*) and those that produce a TRIM5CypA chimera by alternative splicing (*TRIM5^CypA^*). In contrast, we have yet to identify an SIV strain that is sensitive to alleles with a Q at position 339 (*TRIM5^Q^*); thus, it seems likely that animals bearing *TRIM5^Q^* alleles (particularly *TRIM5^Q/Q^* homozygotes) permitted initial spread and adaptation of SIVsm to rhesus macaques, potentially facilitating acquisition of adaptations to overcome other, as yet unidentified genetic barriers encountered in the newly invaded host species. The appearance of the R-to-S and the LPA-to-QQ changes in CA would have opened the way for the virus to spread to a larger percentage of the macaque population, ultimately leading to emergence of pathogenic SIVmac.

None of the viruses tested in this study except HIV-1 were sensitive to the rhesus macaque *TRIM5^Q^* alleles. It is noteworthy that all known human alleles of *TRIM5* have a Q at the homologous position, suggesting that human *TRIM5* may not pose a critical barrier to transmission of SIVsm into human populations; in this regard, it would be interesting to assess the ability of human TRIM5 variants to restrict divergent primary isolates of SIVsm found in regions of endemic infection among African nonhuman primates and to look for correlations between *TRIM5* and susceptibility to HIV-2 in human AIDS cohorts.

An unexpected finding from analysis of the experimental transmission cohorts was the establishment of infection even in animals with restrictive genotypes. In many cases, we found that once animals with restrictive genotypes were infected, viral replication often continued to be suppressed to low levels for many weeks (Figure 4). In nature, lower viremia could reduce the probability that the infected individual will pass the virus on to additional individuals in the new host population, thus extending the role of TRIM5 in preventing emergence beyond the initial cross-species transmission event(s). However, the retrospective analysis of SIVmac emergence does not necessarily address the impact of TRIM5 during natural exposure and transmission, and it is possible that the observed pattern is, at least in part, the consequence of experimental routes of transmission. For example, in many SIV/macaque studies, including the cohorts analyzed here, high titer stocks of virus (TCID_50_ in the range of 1 to 1,000) are used to initiate infections by intravenous injection. Since the effect of restriction by TRIM5α is known to be saturable, it is possible that a large bolus of localized infection manages to initially swamp out restriction and permits virus to seed a large number of virus producing-cells and initiate a transient acute infection. In this regard, experimental intravenous infection of macaques may more closely resemble accidental human exposure to HIV-1 via blood-transfusion or contaminated, hypodermic syringes. It is possible that the effect of TRIM5 could be greater under conditions of natural exposure to virus, such as through sexual contact or fighting; however, we are currently unaware of any studies that address this issue. In recent years, many SIV/AIDS vaccine studies have incorporated low-dose mucosal challenges, to more closely mimic the conditions of sexual transmission. Under such conditions, it is possible that the effect of *TRIM5* genotype on infection will be even more pronounced. Just as historical samples were used for this study, genotyping of appropriate archived samples from completed or ongoing SIV/vaccine studies may also prove useful for evaluating the interactions between *TRIM5* genotype, viral dose, and route of transmission.

Alternatively, there are published reports indicating that *TRIM5* gene expression is interferon-regulated [48],[49]; thus, it is also possible that the full impact of TRIM5 on viral replication in vivo is not manifest until after the first round of infection is well underway. Such a pattern is consistent with the observation that the impact of *TRIM5* genotype in the SIVsmE543-3 infected animals appeared to persist during the weeks following acute infection (Figure 4). The *TRIM5* gene is also known to encode multiple splice-isoforms [50], including mRNA molecules lacking viral specificity encoded by a B30.2/SPRY or CypA domain; however, it is not known whether these are differentially expressed in vivo in response to infection or whether patterns of isoform expression differ between tissues. In vivo studies will be required to determine how *TRIM5* is regulated, whether regulation changes in response to viral infection, and to identify the patterns of *TRIM5* expression in cell types responsible for initial infection and spread in vivo.

The impact of variation in rhesus macaque *TRIM5* has practical implications for preclinical AIDS vaccine research. SIVsmE543 and the related SIVsmE660 serve as heterologous challenge strains for widely used SIVmac239-derived vaccine immunogens in rhesus macaques [38],[51]–[54]. The pronounced effect of *TRIM5* variation on SIVsmE543 infection is likely to confound comparison of vaccinated and control groups, particularly if they are not balanced for restrictive and permissive alleles; this may be especially true for studies with typically small numbers of animals (*n* = 4–6) in each group. This may also be true of SIV strains not examined here, and possibly extends to other SIV/AIDS model organisms including Chinese-origin rhesus macaques and other commonly studied *Macaca* species, such as *M. nemestrina* and *M. fascicularis*. Moreover, vaccines expressing Gag, the target of TRIM5, may be affected by *TRIM5* genotype. This is potentially the case for live-attenuated SIV, where vaccine efficacy is influenced by the degree to which the vaccine strain replicates in the inoculated animal [55]. Finally, as discussed above, the potential effects of viral dose or route of transmission on restriction by TRIM5 remain to be determined.

*TRIM5* variation and susceptibility to infection has been explored in candidate gene studies in HIV/AIDS cohorts, although reported associations were weak [56]–[59]. Recently, a modest but significant association between *TRIM5* genotype and infection in a cohort of SIVmac251-infected macaques was described [24]. While the SIV study did not correlate individual *TRIM5* polymorphisms with viral replication levels, we note that all haplotypes associated with lower viremia in that report were of the *TRIM5^TFP^* class (*TRIM5^CypA^* was excluded from their analysis) [24]. The magnitude of the effect on SIVmac251 (1.3 log) was smaller than we observed with SIVsmE543 or SIVsmE041 (∼2.0 to 3.0 log). This is consistent with the fact that SIVmac251, like its derivative SIVmac239, is the product of years of adaptation in rhesus macaques, while SIVsmE543 and SIVsmE041 have had little or no opportunity, respectively, to adapt to this species [25],[29],[31]. Similarly, lack of a strong association between *TRIM5* variation and infection in HIV/AIDS cohorts may be due to limited variation in human *TRIM5* and the fact that HIV-1 has been spreading in human populations for decades [60],[61]. Our results, particularly when taken in light of the results from HIV/AIDS cohorts and the SIVmac251 cohort, support the conclusion that *TRIM5* primarily governs the transmission of viruses between genetically distinct populations or species and further suggest that the impact of restriction can diminish as a virus spreads and adapts to a new host population.

## Methods

### Ethics Statement

All analyses were performed using archived material, and none of the analyses described involved new animal experiments. Animals at the New England Primate Research Center (NEPRC) were maintained in accordance with standards of the Association for Assessment and Accreditation of Laboratory Animal Care and the Harvard Medical School Animal Care and Use Committee. Animal experiments were approved by the Harvard Medical Area Standing Committee on Animals and conducted according to the principles described in the Guide for the Care and Use of Laboratory Animals. All animals at the NIH were housed in accordance with American Association for Accreditation of Laboratory Animal Care standards, and the NIH investigators adhered to the Guide for the Care and Use of Laboratory Animals prepared by the Committee on Care and Use of Laboratory Animals of the Institute of Laboratory Resources, National Resource Council, and the NIAID Animal Care and Use Committee-approved protocols.

### Plasmids

The SIVmac239-based retroviral vector V1EGFP (gift from Hung Fan, University of California, Irvine, CA) was modified to contain a functional gag-pol ORF. The NarI and SphI sites were used to replace SIVmac239 sequence with that of SIVsmE543 (pGEM-E543 was a gift from Vanessa Hirsch, NIAID, Bethesda, MD). For this purpose the SphI site was introduced by PCR (forward: 5′-CCTAGCAGGTTGGCGCCTGAACAGG-3′, reverse: 5′-GTTATAGCATGCCTCTAGAGGGCGG-3′). A 5′ half of SIVsmE041 was synthesized by GENEART (Regensburg, Germany) with the sequence based on a consensus of sequences obtained by A.K. and S.O. The fragment was engineered to contain NarI and SphI sites for subcloning into V1EGFP. SIVstm/37.16 (gift from Frank Novembre, Emory University, Atlanta, GA) was used for subcloning the SIVstm gag into V1EGFP using the NarI and SbfI sites.

### Primary Cells and Cell Lines

Primary Blood Mononucleated Cells (PBMC) were isolated from heparin- or EDTA-treated rhesus macaque blood samples by density centrifugation with Lymphocyte Separation Medium. PBMC were activated with RPMI/10% FBS containing 50 IU/ml phytohaemagglutinin or 5 mg/ml Concanavalin A for 2 d; afterwards cells were maintained in R10 supplemented with 50 IU/ml interleukin-2.

Crandell-Rees Feline Kidney (CRFK) cells as well as Human Embryonic Kidney 293T/17 (HEK293T/17) cells were obtained from American Type Culture Collection (Manassas, VA) and grown in DMEM/10% FBS. Generation of stable CRFK cell lines was done as previously described in Newman et al., PNAS 103(50), 2006 [15]. For the experiments performed here, new stable CRFK cell lines were made to express N-terminally HA-tagged rhesus *TRIM5* alleles. For this purpose an HA tag was added by PCR (forward: 5′-GCGGCCGCATGGCTTCTGGAATC-3′; reverse: 5′-CCACCGGTGGCTCAAGCGTAGTCTGGGACGTCGTATGGGTAGCCGCCAGAGCTTGGTGAGCACAGAG-3′) and the NotI and AgeI sites were used for subcloning into pQCXIN (BD Biosciences, Franklin Lakes, NJ). Stable cell lines were maintained in DMEM/10% FBS supplemented with 0.5 mg/ml G418. All cultured cells were maintained at 37°C with 5% CO_2_.

### Viruses

All single-cycle SIV viruses were produced in HEK293T/17 cells by cotransfection of the appropriate V1EGFP-SIV plasmid and pVSV-G (Clontech Laboratories, Mountain View, CA), using the GenJet transfection system (SignaGen; Ijamsville, MD). The single-cycle HIV-1 virus stock was made by cotransfection of pNL43DenvGFP and pVSV-G. Single-cycle HIV-2 was made by cotransfection of pHIV-2(ROD)GFP and pVSV-G. Culture supernatants containing the single-cycle, GFP/EGFP expressing, VSV-G-pseudotyped virions were titered on untransfected CRFK cells; supernatant volumes resulting in approximately 30% GFP/EGFP^+^ CRFK cells were used for infectivity assays on the stably transfected cell lines expressing the various rhesus *TRIM5* alleles.

### Infectivity Assays

Stable CRFK cells were seeded at a concentration of 5×10^4^ cells per well in 12-well-plates and infected with the appropriate amount of VSV-G pseudotyped, single-cycle, GFP/EGFP expressing viruses. All infections were done in triplicate. After 3 d, expression of GFP/EGFP was analyzed by fluorescence-activated cell sorting (FACS) performed on a FACSCalibur™ flow cytometer (BD, Franklin Lakes, NJ), and data were analyzed using FlowJo (Tree Star, Inc., Ashland, OR).

### PCR (Site-Directed Mutagenesis, Genotyping, Sequencing)

The Q^89^Q^90^ to LPA and S97R mutants of SIVmac239 capsid were made by site-directed mutagenesis on a SIVmac239 5′ plasmid (Q^89^Q^90^ to LPA: forward: 5′-GCAGATTGGGACTTGCAGCACCCAATACCAGGCCCCTTACCAGCGGGACAACTTAGGGAGCCGTCAGG-3′; reverse: 5′CCTGACGGCTCCCTAAGTTCTCCCGCTGGTAAGGGGCCTGGTATTGGGTGCTGCAAGTCCCAATCTGC-3′) (S97R: forward: 5′-CCCACAACCAGCTCCACAACAAGGACAACTTAGGGAGCCGAGGGGATCAGATATTGCAGGAAC-3′; reverse: 5′-GTTCCTGCAATATCTGATCCCCTCGGCTCCCTAAGTTCTCCTTCTTCTGGAGCTGGTTGTGGG-3′).

The IPA-to-Q**^89^**Q**^90^** mutants of SIVsmE041 were made by site-directed mutagenesis on a SIVsmE041 5′ plasmid (IPA-to-QQ: forward: 5′-CCACAGCCAGGTCCACAACAAGGACAACTTAGAGACCCGAGAGG-3′; reverse: 5′-CCTCTCGGGTCTCTAAGTTGTCCTTGTTGTGGACCTGGCTGTGG-3′). For all mutants, NarI and SphI sites were used for subcloning into V1EGFP.

*TRIM5* genotypes of rhesus macaques were determined by isolation of genomic DNA from PBMC using the QIAamp DNA Blood Mini kit (QIAGEN, Valencia, CA) and direct sequencing of a PCR fragment (forward: 5′-CAGTGCTGACTCCTTTGCTTG-3′; reverse: 5′-GCTTCCCTGATGTGATAC-3′) of the C-terminal B30.2/SPRY domain of *TRIM5*. The following PCR protocol was implemented: 1 min at 94°C initial denaturation, 15 s at 94°C denaturation, 30 s at 55°C annealing, 1 min at 68°C extension, 10 min at 68°C final extension; steps 2 through 4 were repeated for 30 cycles. PCR fragments were sequenced by Retrogen (San Diego, CA) and data were analyzed with the Codoncode software (Codoncode Corporation, Dedham, MA).

Viral RNA was extracted from blood plasma with the High Pure Viral RNA kit (Roche Diagnostics Corporation, Indianapolis, IN) and partial capsid sequence was determined by direct sequencing of an RT-PCR fragment (forward: 5′-GAAGCTTGCCACCATGGGCGCGAGAAACTC-3′; reverse: 5′-CCTCTCTGTTGGACTGCTGC-3′).

### Immunoblotting

Stable CRFK cells expressing the various HA-tagged *TRIM5* alleles were seeded at a density of 5×10^4^ cells per well in a 6-well-plate. After 48 h, cells were lysed in M-PER reagent (Pierce Biotechnology, Rockford, IL), and total protein concentration of each lysate was determined by measurement of A280 with a NanoDrop spectrophotometer (Thermo Fisher Scientific, Waltham, MA). Equal amounts of total protein were separated by SDS/PAGE and HA-tagged TRIM5 proteins were detected with rat monoclonal Anti-HA-Peroxidase High Affinity antibody (Roche Diagnostics Corporation, Indianapolis, IN). b-actin was detected with mouse monoclonal beta Actin-HRP antibody (Abcam Inc., Cambridge, MA).

### Experimental Infections

Forty-four rhesus macaques of Indian origin were infected with SIVsmE543-3 either intravenously (*n* = 35) or intrarectally (*n* = 9) with a TCID 50% ranging from 1 to 1,000. There was no statistically significant difference in viral RNA levels relative to route of inoculation (unpublished data). None of the animals were treated or vaccinated prior to infection. The majority of the animals (*n* = 41) were inoculated with a cell-free virus stock generated by infection of pigtailed macaque PBMC with virus produced by transfection of CEMx174 cells with the SIVsmE543-3 molecular clone.

### Blood Plasma Viral Loads

The quantification of plasma viral loads was described previously in Hirsch et al. [29]. Briefly, viral RNA loads were determined by real-time reverse transcriptase-PCR (RT-PCR). For this purpose, viral RNA from plasma was serially diluted and used as a template in a RT-PCR reaction, together with known amounts of pSG83 as an internal control template. Results were normalized to the volume of plasma extracted and expressed as SIV RNA copies per milliliter of plasma.

## Supporting Information

Figure S1 **Production of VSV-G pseudotyped, single-cycle SIV for restriction assays.** (A) The modified V1EGFP vector, as described in Methods. (B) pVSV-G vector. (C) Single cycle SIVmac239 titration on parental CRFK cells (TRIM5-null). (D) Single-cycle SIVmac239QQ->LPA. (E) Single cycle SIVmac239S->R. (F) Single cycle SIVsmE543-3. (G) Single-cycle SIVsmE041. Virions were produced by transient co-transfection of a vector expressing viral proteins (A) and a second vector expressing the Vesicular Stomatitis Virus G-protein (B). The viral vector also produces messenger RNA containing a transducible enhanced Green Fluroescent Protein (eGFP) ORF in place of the viral nef gene (green box); in the subsequent round of infection, the reporter RNA is reverse transcribed and integrated into the infected cell. Infection is then monitored by flow-cytometry to count eGFP-positive cells as a percent of total live cells (C–G).
(8.33 MB TIF)

## Abbreviations

CA: capsid protein
CypA: cyclophilin-A
NTD: N-terminal domain
RF: restriction factor
SIVs: Simian immunodeficiency viruses
SIVmac: Simian immunodeficiency viruses of rhesus macaques
SIVsm: Simian immunodeficiency viruses of sooty mangabeys
SIVstm: Simian immunodeficiency viruses of stump-tailed macaques
