# Supplementary figures and images for: TRIM5 Suppresses Cross-Species Transmission of a Primate Immunodeficiency Virus and Selects for Emergence of Resistant Variants in the New Species
Source: PLoS Biol. 2010 Aug 24;8(8):e1000462. doi: 10.1371/journal.pbio.1000462 (PMC2927514; doi:10.1371/journal.pbio.1000462)

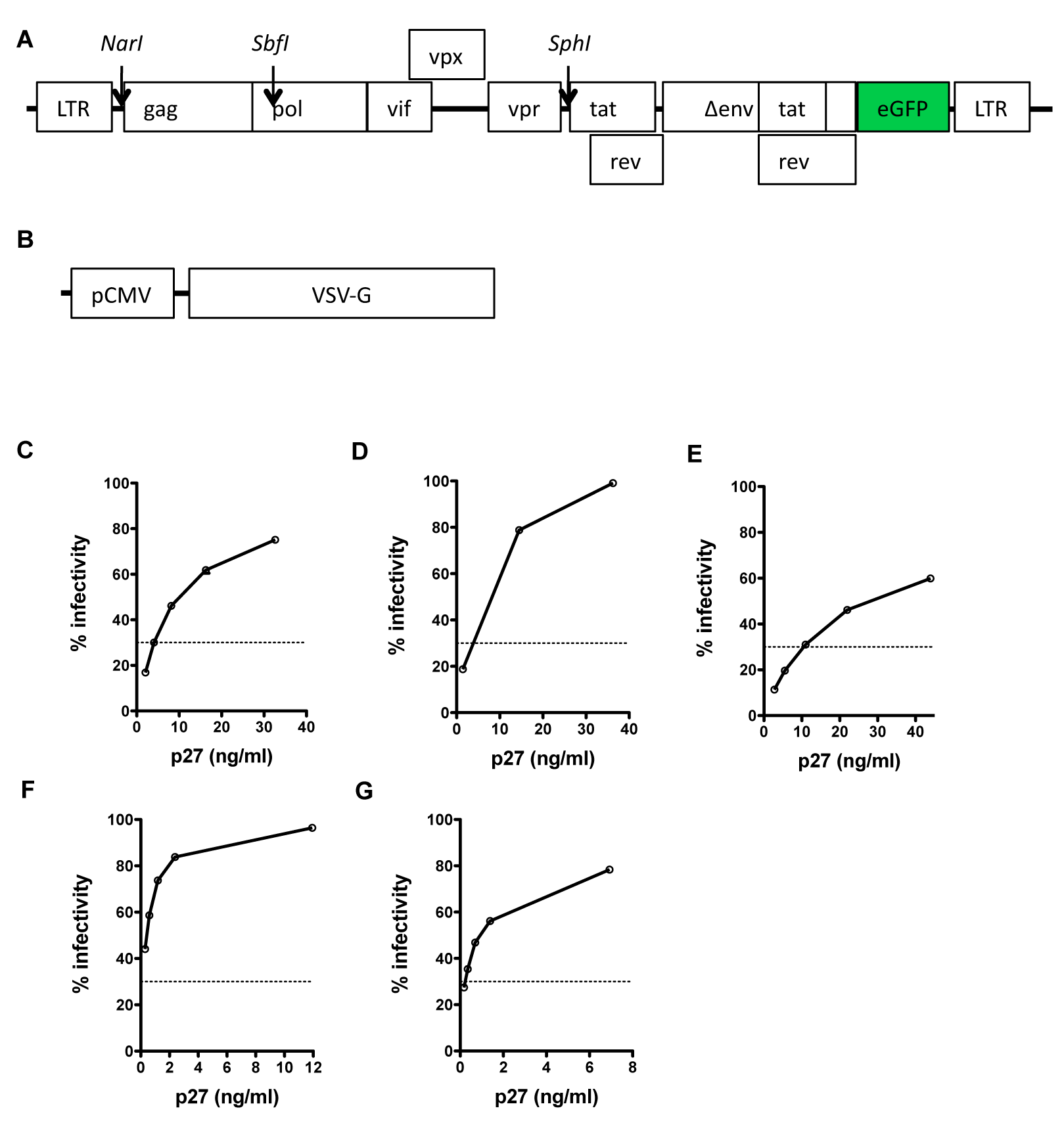

Supplement: Figure S1 — Production of VSV-G pseudotyped, single-cycle SIV for restriction assays. (A) The modified V1EGFP vector, as described in Methods. (B) pVSV-G vector. (C) Single cycle SIVmac239 titration on parental CRFK cells (TRIM5-null). (D) Single-cycle SIVmac239QQ->LPA. (E) Single cycle SIVmac239S->R. (F) Single cycle SIVsmE543-3. (G) Single-cycle SIVsmE041. Virions were produced by transient co-transfection of a vector expressing viral proteins (A) and a second vector expressing the Vesicular Stomatitis Virus G-protein (B). The viral vector also produces messenger RNA containing a transducible enhanced Green Fluroescent Protein (eGFP) ORF in place of the viral nef gene (green box); in the subsequent round of infection, the reporter RNA is reverse transcribed and integrated into the infected cell. Infection is then monitored by flow-cytometry to count eGFP-positive cells as a percent of total live cells (C–G). (8.33 MB TIF) [file pbio.1000462.s001.tif]
